# A computational study of organic molecular crystals for photocatalytic water splitting

**DOI:** 10.1039/d6fd00050a

**Published:** 2026-07-20

**Authors:** James D. Green, Hong Wang, Andrew I. Cooper, Daniel G. Medranda, Jenny Nelson, Kim E. Jelfs

**Affiliations:** a Department of Chemistry, Molecular Sciences Research Hub, Imperial College London London W12 0BZ UK j.green@ic.ac.uk; b Department of Chemistry, University of Liverpool Liverpool L69 7ZD UK; c Department of Physics, Imperial College London London UK

## Abstract

Organic crystalline materials are potential candidates for photocatalytic overall water splitting (OWS). Although organic crystals have been heavily investigated for application in organic electronics, such as organic light-emitting diodes (OLEDs) and solar cells, there have been comparatively fewer studies into OWS in these materials. A major challenge is the large number of electronic and structural criteria that must be met for a material to make a viable OWS photocatalyst. Optical absorption, reduction and oxidation potentials and charge-transport properties are among the key considerations, and these are influenced both by molecular structure and the solid-state packing arrangement, making computational modelling challenging. Here, we investigate a series of known organic electronic materials using periodic density functional theory (DFT) and compare their calculated electronic properties of optical absorption and reduction and oxidation potentials with literature experimental data. We also perform a series of gas-phase molecular calculations, which show a good agreement with both the literature data and periodic DFT calculations for the optoelectronic properties of the systems studied, showing that gas-phase molecular calculations can be used to screen organic crystals for OWS at a reduced computational cost.

## Abstract 2

Computationally expensive periodic DFT calculations are not necessary to screen the electronic properties of molecular crystals for photocatalytic water splitting, as fast single-molecule calculations are shown to provide similar accuracy.

## Graphical abstract

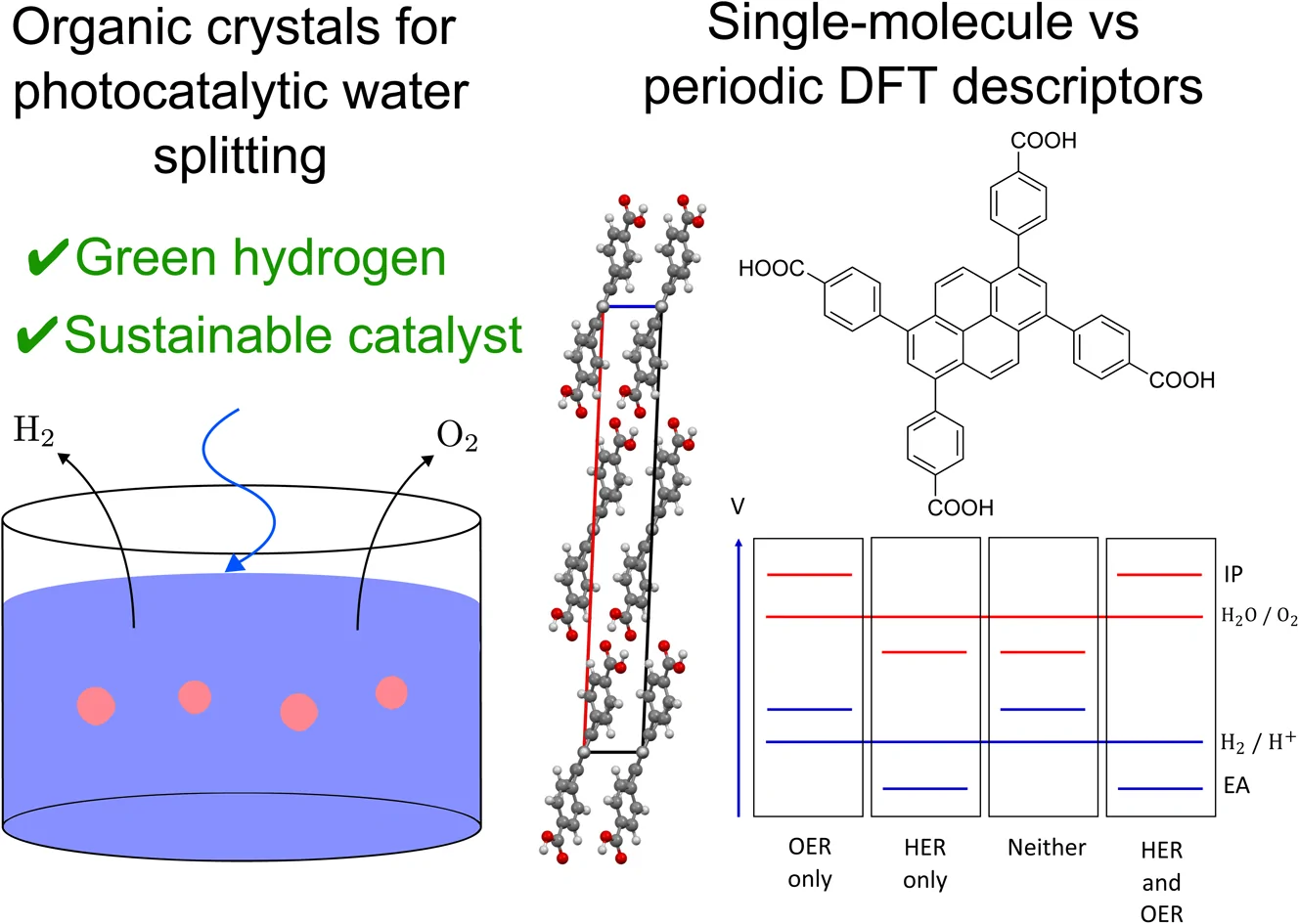

## Introduction

Recent years have seen a progressive shift towards organic materials in optoelectronic applications, not only offering a more sustainable and cost effective alternative to conventional inorganic materials, but also offering new functionality and tunability.^1–9^ For example, organic light-emitting diodes (OLEDs) has taken over the market for high-end displays, and organic photovoltaics (OPV) have found a useful niche for indoor devices among other applications.^1,2,4,6,8,10–12^ Photocatalysts are yet another industrially relevant class of optoelectronic materials, spanning a wide range of different reactions from removing air pollutants to producing chemical feedstocks and fuels using sunlight.^9,13–15^ Photocatalytic processes have also benefitted from ever-improving organic catalysts.^9^ One highly promising photocatalytic process for the future energy economy is the solar-driven overall water splitting (OWS) reaction, where water is converted into hydrogen and oxygen by the aid of sunlight and a photocatalyst, without requiring any sacrificial agents.^14,16–18^ OWS consists of two half-reactions: the hydrogen evolution reaction (HER), where 2H^+^ is reduced to H_2_, and the oxygen evolution reaction (OER), where H_2_O is oxidised to 
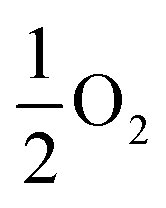
 and 2H^+^.

The best currently known photocatalysts for OWS are crystalline inorganic materials, for example NaTaO_3_,^19^ Ga_2_O_3_,^20^ and SrTiO_3_^21^ have all been widely studied. There have also been sustained efforts to find competitive organic photocatalysts for OWS, however, thus far the solar hydrogen and oxygen production rates of organic materials have fallen short of those demonstrated by inorganic materials.^18,22–25^ Despite this, some promising materials have been identified, with efforts focused primarily on extended organic materials, such as carbon nitrides^17,23,26^ and 2D/3D covalent organic frameworks.^18,25^ More recently, HER and OER have also been demonstrated in organic molecular crystals, although OWS has yet to be achieved in these systems (and the rates of HER and OER seen thus far are relatively low).^24,27–29^ One of the fundamental challenges is that very few organic materials have the correct energetic properties to simultaneously catalyse HER and OER, although Z-scheme systems in which two complementary materials are combined to simultaneously catalyse OER and HER offer a solution to this challenge.^30,31^ Given the chemical and structural diversity of organic molecular crystals, and also that comprehensive studies of such materials for OWS are relatively sparse in the literature, this prompted a focused computational investigation into the electronic properties of such materials for OWS.

To propose suitable materials for OWS with the necessary electronic properties, we must examine the initial steps of the OWS reaction, namely photoexcitation of the catalyst, charge separation, charge-carrier transport (to the interface of the catalyst and water) and charge injection (of electrons and holes into protons and water respectively). Firstly, it is fairly obvious that many materials can be ruled out as photocatalysts based on their inability to absorb light in the solar spectrum. Secondly, even after successful photoexcitation, it is by no means a given that sufficient charge separation also occurs. Thirdly, provided that charge-separation does occur, sufficient numbers of charge carriers will need to make it to the surface of the photocatalyst material before recombining. Fourth and finally, the electrochemical potential of the photogenerated electrons and holes still needs to be sufficient to allow for the injection of charges into the protons and water. Therefore the electronic properties and photophysics of the photocatalyst material itself is of primary importance to allow adequate HER and OER performance.^14,16–18^

The fourth step, injection of charges, is more complicated, as in reality it is necessary to use a co-catalyst that acts as an intermediary between the catalyst and protons/water to reduce the activation energy of these charge transfer reactions and allow OWS to occur at a reasonable rate.^17^ In practice, separate HER and OER cocatalysts are used to accept electrons and holes respectively from the photocatalyst and inject them into the protons and water, however, consideration of cocatalysts does not ultimately change the energetic criteria that electrons and holes must provide sufficient driving force for the OER and HER reactions.^17^ Here, we focus on the optical absorption and reduction/oxidation potentials of molecular electronic materials. We will also briefly introduce charge-transport in molecular crystals but will leave discussion of charge separation aside.

Fortunately, theoretical models and computational calculations allow us to interrogate the fundamental electronic properties of materials. Quantum chemical calculations using density functional theory (DFT) are a particularly popular approach and can provide accurate insight into such properties.^32–35^ These calculations are grouped into three main types based on the description of the chemical system: (i) solid-state DFT calculations describing the system as a bulk material using periodic boundary conditions (PBC);^36,37^ (ii) molecular DFT calculations describing the system as an isolated molecule or molecules,^38^ either in the gas-phase or in solution; and calculations of materials described by large clusters of molecules not imposing periodic boundary conditions, but instead using interacting quantum mechanics (DFT) and molecular mechanics regions (QM/MM).^39,40^

With relevance to OWS, three key types of descriptors can be calculated for materials that relate to their performance and suitability as photocatalysts.

### Optical absorption

The excited states of a material can be calculated using linear response methods such as time-dependent DFT (TD-DFT) in the approximation of vertical transitions. The energy of the first excited state (S_1_) is often used as a reference point, as this is the lowest energy photon that can be absorbed *via* an electronic transition in the material.^41–43^ Additionally, the full UV-visible spectrum of the material may be simulated taking into account all calculated states and their oscillator strengths, providing a clearer picture of which electronic states will be responsible for absorption across the UV-visible spectrum, especially important when S_1_ has a vanishingly small oscillator strength.^43–45^

### Reduction and oxidation potentials

Ionisation potential (IP) and electron affinity (EA) are key to determining whether the photogenerated holes and electrons have sufficient electrochemical potential to drive OER and HER respectively. In the vertical approximation, *i.e.* assuming that the process is instantaneous and the geometric structure and one-electron wavefunctions are unchanged during the reaction, the IP and EA can be directly calculated from the negative of the valence band maximum (VBM) and conduction band minimum (CBM) positions respectively correcting for the electrostatic potential of the crystal relative to vacuum in a bulk system. In the molecular picture it is even simpler, the vertical IP and EA are the negative of the highest occupied molecular orbital (HOMO) and lowest unoccupied molecular orbital (LUMO) energies respectively, invoking Koopman's theorem.^46^ The IP must be greater than the electrochemical potential of the water oxidation reaction (
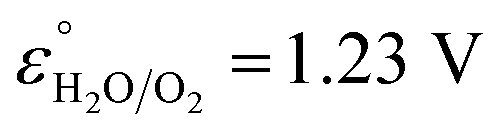
 relative to the standard hydrogen electrode, 
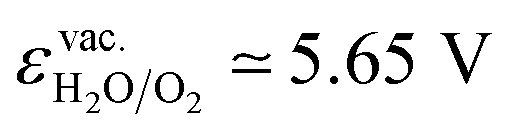
 relative to the vacuum level) and the EA must be lower than the electrochemical potential of the proton reduction reaction (
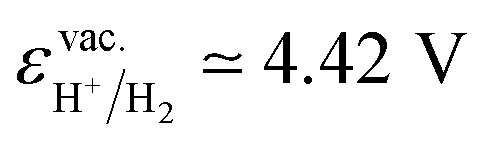
 relative to the vacuum level).^47–50^

### Charge transport

Though we do not numerically evaluate the charge transport properties of the organic molecular crystals here, as an aside we will briefly introduce some concepts of charge transport.

The effectiveness of charge carrier (electron and hole) transport through a material is usually quantified by the carrier mobility, *µ*, normally measured in units of cm^2^ V^−1^ s^−1^. *µ* is an empirical quantity, but can be estimated by first-principles simulations.^51,52^ These simulations are based on either a delocalised, band-like model or a localised, hopping-like model of charge transport. The choice of model depends on the nature of the system under investigation, where for very delocalised periodic materials the band-like model is most appropriate, and for very localised, molecular systems especially those with low translational symmetry, the molecular hopping-like model is most appropriate. These two models, however, are limiting cases and in many organic materials, aspects of either model apply.^53^

In the case of band-like transport, charge carriers are completely delocalised across the material and move continuously in bands. Band-like transport is hindered by structural defects and thermal vibrations (phonons) resulting in a negative temperature coefficient, *i.e.* decrease in mobility with increasing temperature. *µ* can be shown to depend on the effective carrier masses m* at the band edges and scattering times *τ* due to thermal noise and defects according to the band-like model 
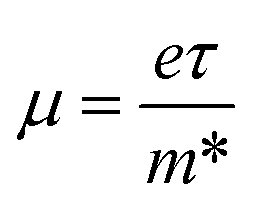
.^54–58^

In the opposite extreme, the wavefunctions of charge carriers are completely localised to individual sites in the material and transport occurs *via* discrete, thermally activated hops between sites, which in this case are individual molecules. This hopping-like transport is characterised by an initial increase in mobility with increasing temperature up until an optimum after which mobility decreases again. The hopping-rate *Γ*_*ij*_ between individual pairs of molecules *i* and *j*, a fundamental property, can be expressed in a Marcus model 
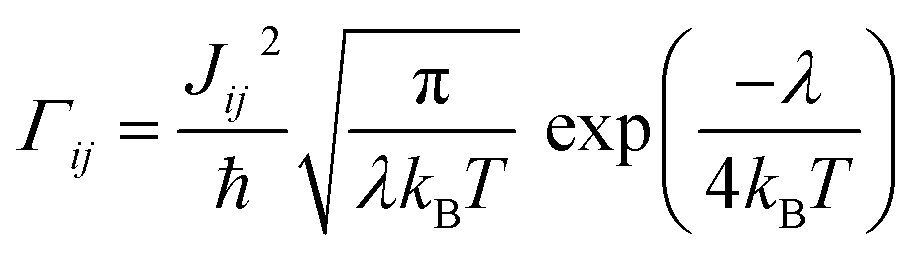
, where *λ* is the reorganisation energy, usually considered to be an intrinsic molecular property, *J*_*ij*_ the transfer integral, a property linked to pairs of molecules, *k*_B_ the Boltzmann constant, ℏ the reduced Planck constant and *T* the temperature. This model assumes all molecules are identical, there is no applied electric field and that the carrier concentration is low. The mobility can then be simulated by calculating *Γ*_*ij*_ for all pairs of molecules in the material and either directly solving the master equation, applying Monte Carlo simulations or approximated from the diffusion coefficients *D*_*α*_ in each cell direction *α* using the Einstein relation 
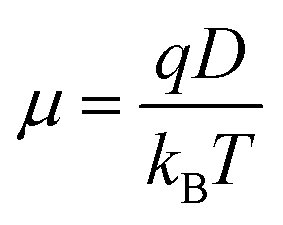
.^51–54,59–61^

The quantities m* and *τ* (band-like model) and *J*, *λ* and *Γ* (hopping-like model) can be theoretically predicted by DFT calculations.^51,53^ Both m* and *J* are shown to weakly correlate with the experimentally measured mobility—a decrease in m* or increase in *J* is usually expected to result in a higher *µ*. Furthermore, for molecular systems, the value of 2*J*/*λ*, a qualitative approximation for the electron–phonon coupling strength, can be used to roughly categorise transport as band-like when 2*J*/*λ* ≫ 1, or hopping-like when 2*J*/*λ* ≪ 1.^53^

Here, we consider the five organic molecular crystals presented in Fig. 1 that have all been previously investigated for (opto)electronic devices, namely: rubrene, 4,4′,4″,4‴-(pyrene-1,3,6,8-tetrayl)tetrabenzoic acid (TBAP), perylenetetracarboxylic dianhydride (PTCDA), dimethyl perylenetetracarboxylic diimide (Me-PTCDI) and 5,10,15,20-tetra(4-pyridyl)-porphine (TPyP).^24,28,29,62–67^ Of these molecules, one is an acene (rubrene), one is pyrene-based (TBAP), two are perylene-based (PTCDA, Me-PTCDI) and one is a porphyrin. The pyrene- and perylene-based molecules (TBAP, PTCDA, Me-PTCDI) and their derivative compounds have also been of interest for photocatalytic HER and OER.^24,28,29^ We apply two of the aforementioned DFT-based descriptors, optical absorption and reduction/oxidation potentials, to these five organic molecular crystals and discuss their potential for OWS. Furthermore, we compare descriptors calculated using solid-state calculations using periodic boundary conditions to less computationally demanding molecular calculations to test the possibility of high-throughput screening candidate organic molecular crystals for OWS using less intensive calculations.

**Fig. 1:**
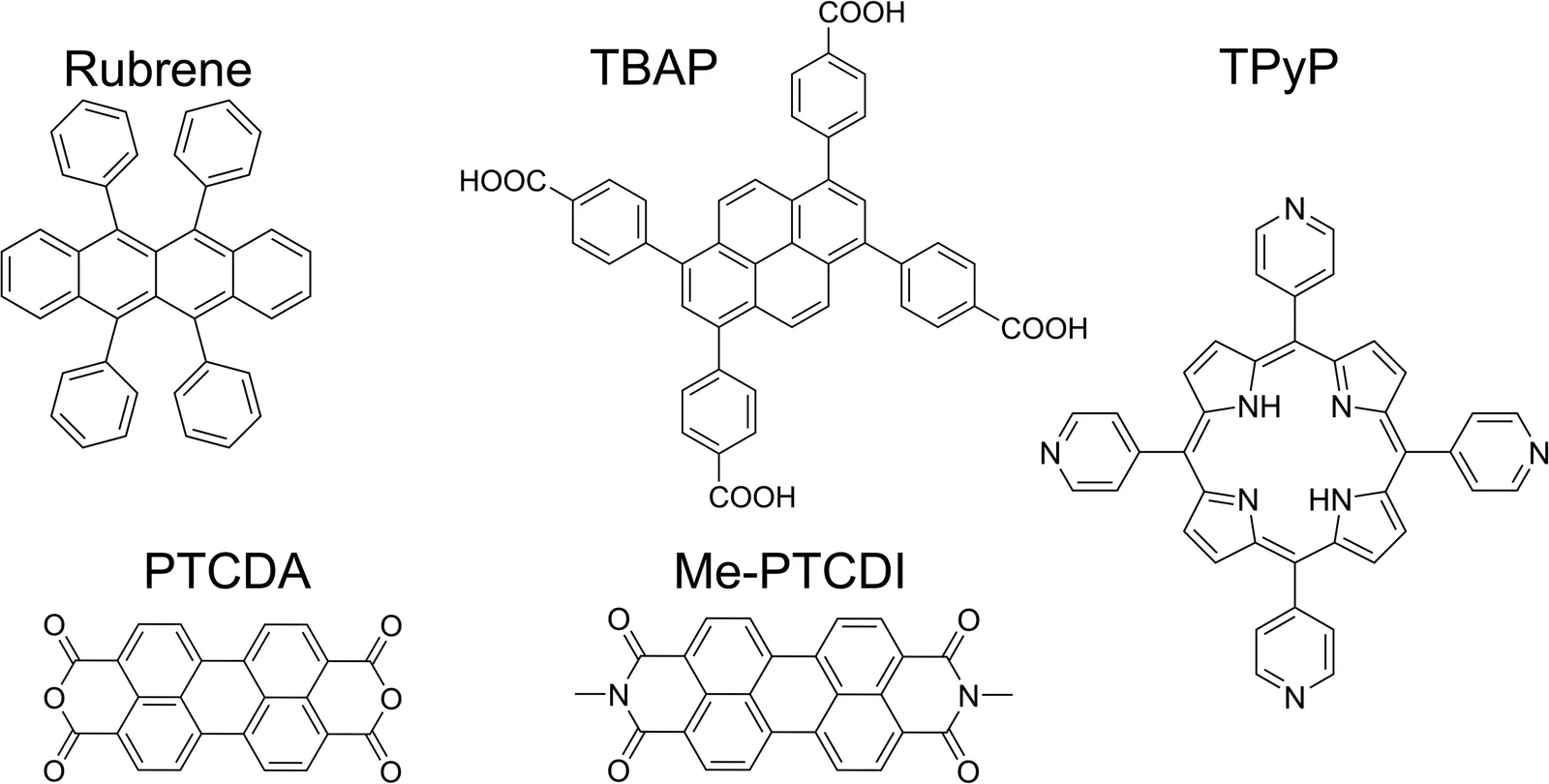
The five molecular crystals studied here: rubrene, 4,4′,4″,4‴-(pyrene-1,3,6,8-tetrayl)tetrabenzoic acid (TBAP), perylenetetracarboxylic dianhydride (PTCDA), dimethyl-perylenetetracarboxylic diimide (Me-PTCDI) and 5,10,15,20-tetra(4-pyridyl)-porphine (TPyP).

## Methods

### Computational crystal structures

The following X-ray crystal structures were taken from the Cambridge Structural Database with reference codes and space groups in parentheses: rubrene (QQQCIG01, *Cmca*), TBAP (MUDSIQ, *C*2/*m*), PTCDA (SUWMIG02, *P*2_1_/*n*), Me-PTCDI (DICPAG, *P*2_1_/*c*) and TPyP (COWSIR, *C*2/*c*).^68^ For each of the structures, the primitive cell and atomic positions were relaxed to their theoretically optimal values using periodic DFT in the Vienna *Ab initio* Structure Package version 6.5 (VASP)^36^ on the Archer2 UK national supercomputer.^69^ For each structure, a sufficiently large *k*-point mesh, using the Monkhorst–Pack scheme, was selected such that the total energy with respect to increasing the number of *k*-points had converged to within 1 eV per atom. The number of *k*-points in respective *a* × *b* × *c* cell directions used for rubrene, TBAP, PTCDA Me-PTCDI and TPyP were 2 × 2 × 2, 3 × 3 × 3, 3 × 3 × 3, 3 × 3 × 3, and 2 × 2 × 2 respectively. The theoretical model employed the Perdew–Burke–Ernzerhof (PBE) density functional with the damped dispersion correction of Becke, Johnson and Grimme (for non-covalent interactions) and used a plane-augmented-wave (PAW) basis with an energy cutoff (ENCUT) of 650 eV.^70,71^ The calculations were deemed to have converged to a relaxed structure when the force acting on all atoms fell below 0.01 eV Å^−1^. The root mean squared deviation of atomic positions for a cluster of 30 molecules (RMSD_30_) between the experimental and DFT relaxed crystal structures calculated in Mercury 2024.1.0^72^ were below 0.3 Å for all molecular crystals using a distance tolerance of 20% and angle tolerance of 20°. The relaxed crystal structures are shown in Fig. 2. Further details of structural relaxations can be found in the SI Sections S1 and S2.

**Fig. 2:**
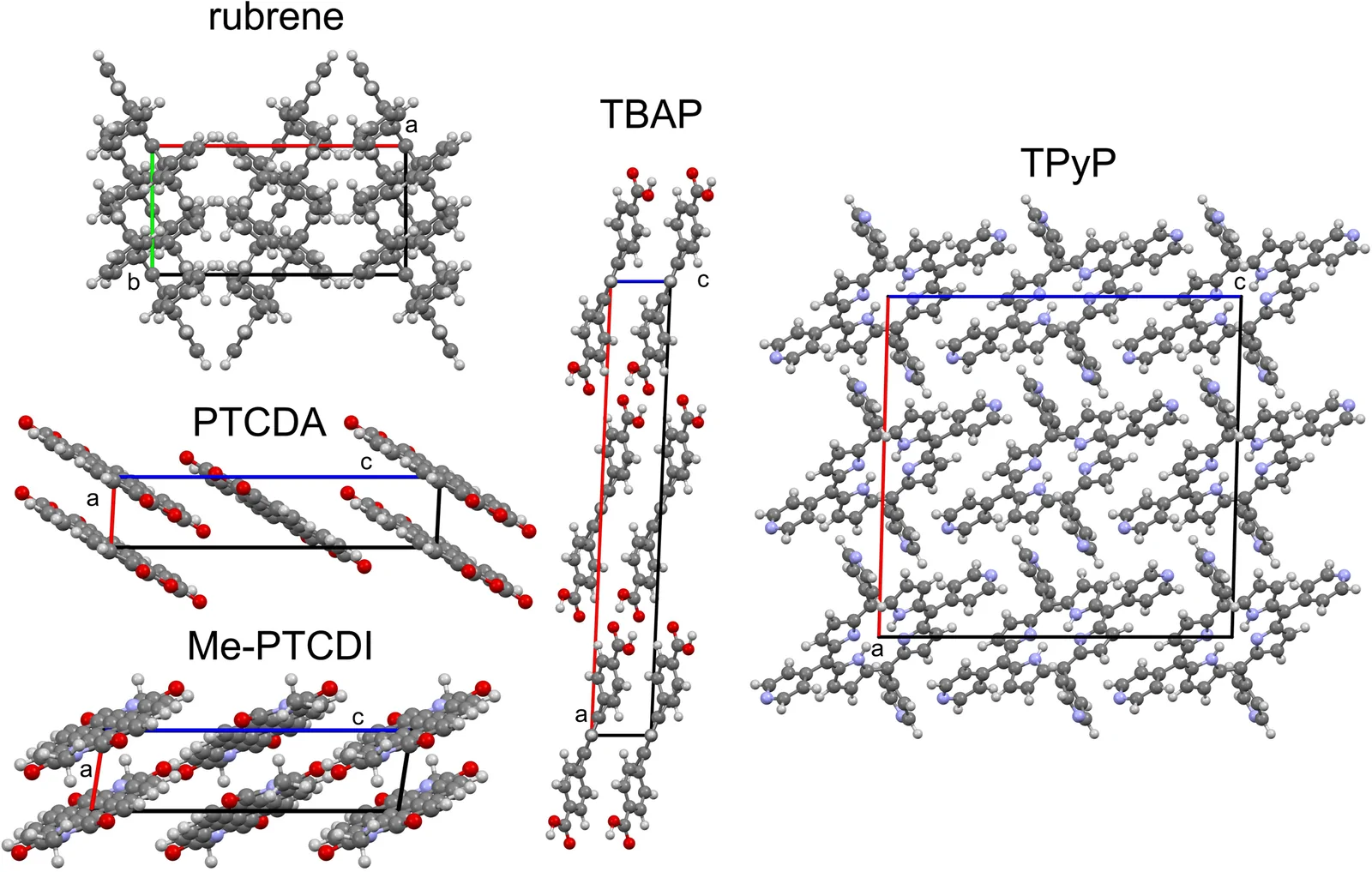
DFT relaxed crystal structures of five organic crystals: (a) rubrene (viewed perpendicular to *c* axis), (b) TBAP (viewed perpendicular to *b* axis), (c) PTCDA (viewed perpendicular to *b* axis), (d) Me-PTCDI (viewed perpendicular to *b* axis), (e) TPyP (viewed perpendicular to *b* axis). C atoms coloured in grey, H in white, N in blue and O in red. Figure produced using Mercury 2024.1.0.^72^

### IP and EA calculations

The computational band structures of bulk crystals were simulated with VASP^36^ on the PBE-D3 relaxed primitive cells using the HSE06 hybrid density functional and ENCUT of 650 eV. Gamma-centred *k*-point meshes of 3 × 3 × 3, 2 × 2 × 4, 4 × 2 × 1, 4 × 2 × 2, and 2 × 2 × 1 points were used for rubrene, TBAP, PTCDA, Me-PTCDI and TPyP respectively. These meshes were chosen by increasing the number of *k*-points in each cell direction until the HSE06 band gap, CBM and VBM at *Γ* had all converged to within 1 × 10^−3^ eV of the value with the largest mesh. The valence band maximum (VBM) and conduction band minimum (CBM) were extracted from the band structures – rubrene has a direct band gap of 1.62 eV whereas TBAP, PTCDA, Me-PTCDI and TPyP have indirect band gaps of 2.26 eV, 1.83 eV, 1.56 eV, and 2.11 eV respectively.

Vacuum slabs were constructed for the organic crystals with a bulk thickness of ∼20 Å and vacuum gap of 40 Å starting from the relaxed cells, similar to the approach of ref. 73 and 74. The electrostatic potential was calculated by a self-consistent calculation on the vacuum slabs with HSE06/ENCUT = 650 eV. The surface dipole method was then used to correct the VBM and CBM for the electrostatic potential inside the bulk crystal to give ionization potentials (IP) and electron affinities (EA) for each crystal in the vertical approximation: IP = *ε*_SD_ − *ε*_VBM_, EA = IP + *ε*_g_ where *ε*_SD_ is the surface dipole energy, *ε*_VBM_ is the VBM energy and *ε*_g_ is the band gap. Further details of the vacuum slab calculations can be found in the SI Sections S1 and S4 and band structures in SI Section S5. These calculations were performed on Archer2.^69^

### Excitation energies of bulk systems

The periodic time-dependent density functional perturbation theory (TD-DFPT) method implemented in the CP2K program version 2024.3 (CP2K) on Archer2^69^ using the Tamm–Dancoff approximation was used to calculate the excited states of supercells of the molecular crystals.^75,76^ The relaxed unit cells were expanded to give supercells with minimum lattice constants of 14 Å for TD-DFPT calculations containing 560, 312, 608, 592, 368 atoms respectively for rubrene, TBAP, PTCDA, Me-PTCDI and TPyP. The first ten roots of the excited state Hamiltonian were calculated which is a sufficiently large number to ensure that the correct S_1_ state was captured for all molecules due to state energy reordering. The M06-2X^77^ and *ω*B97x^78^ functionals were employed with a Gaussian augmented-plane-wave basis (GAPW) using the DZVP-MOLOPT valence basis and GTH-PBE pseudopotential (for core electrons) for all atoms. Input files for CP2K were generated using the MultiWFN package.^79,80^ Further detailed of CP2K TD-DFT calculations can be found in the SI Table S7.

### Molecular excitation and orbital energies

DFT calculations in Orca 6.0^38,81–85^ were performed for molecular HOMO/LUMO energies using a range of functionals (PBE, B3LYP, *ω*B97x, LC-PBE and M06-2X)^70,77,78,86,87^ and the def2-TZVP basis set. The excited states of single molecules were subsequently calculated using standard TD-DFT in Orca 6.0 using the same functionals and basis set.^81,88^ The first ten roots of the excited state Hamiltonian were calculated.

## Results and discussion

### Optical gap

The first excited state (S_1_) energies of the five compounds calculated by periodic TD-DFPT using M06-2X are shown in Table 1 and Fig. 3. These calculations predict that all molecules have an optical gap within the visible region (400–700 nm). When compared to the optical gaps taken from thin-film UV-visible absorption spectra,^89–92^ the overall accuracy of periodic TD-DFPT (M06-2X) is reasonably good for all molecules, predicting optical gaps within 0.2 eV of the experimental values for rubrene, TBAP, PTCDA and Me-PTCDI and within 0.3 eV for TPyP. However, as evident from Fig. 3, the trend in the S_1_ energies from periodic M06-2X deviates from that of experiment, as TPyP, which has the lowest experimental S_1_ energy, is predicted to have the second lowest by periodic M06-2X. TD-DFPT calculations were repeated with periodic *ω*B97x which correctly reproduces the experimental trend in the S_1_ energies at the cost of some accuracy. Here the S_1_ energy of TPyP is stabilised by around 0.2 eV, but conversely the S_1_ energies of all other molecules are destabilised by a similar amount in *ω*B97x relative to M06-2X.

**Table 1:** S_1_ energies calculated for a series of organic molecular crystals in TD-DFPT using periodic M06-2X/DZVP and periodic *ω*B97x/DZVP. The experimental optical gaps measured in the solid state are also included with their corresponding literature reference provided

Molecule	S_1_ (M06-2X)/eV	S_1_ (*ω*B97x)/eV	S_1_ (exp.)/eV	Ref.	Error (M06-2X)/eV	Error (*ω*B97x)/eV
Rubrene	2.37	2.63	2.32	89	0.05	0.31
TBAP	2.93	3.00	2.74	90	0.18	0.26
PTCDA	2.40	2.53	2.20	91	0.20	0.33
Me-PTCDI	2.22	2.44	2.15	91	0.07	0.29
TPyP	2.23	2.06	1.93	92	0.30	0.12

**Fig. 3:**
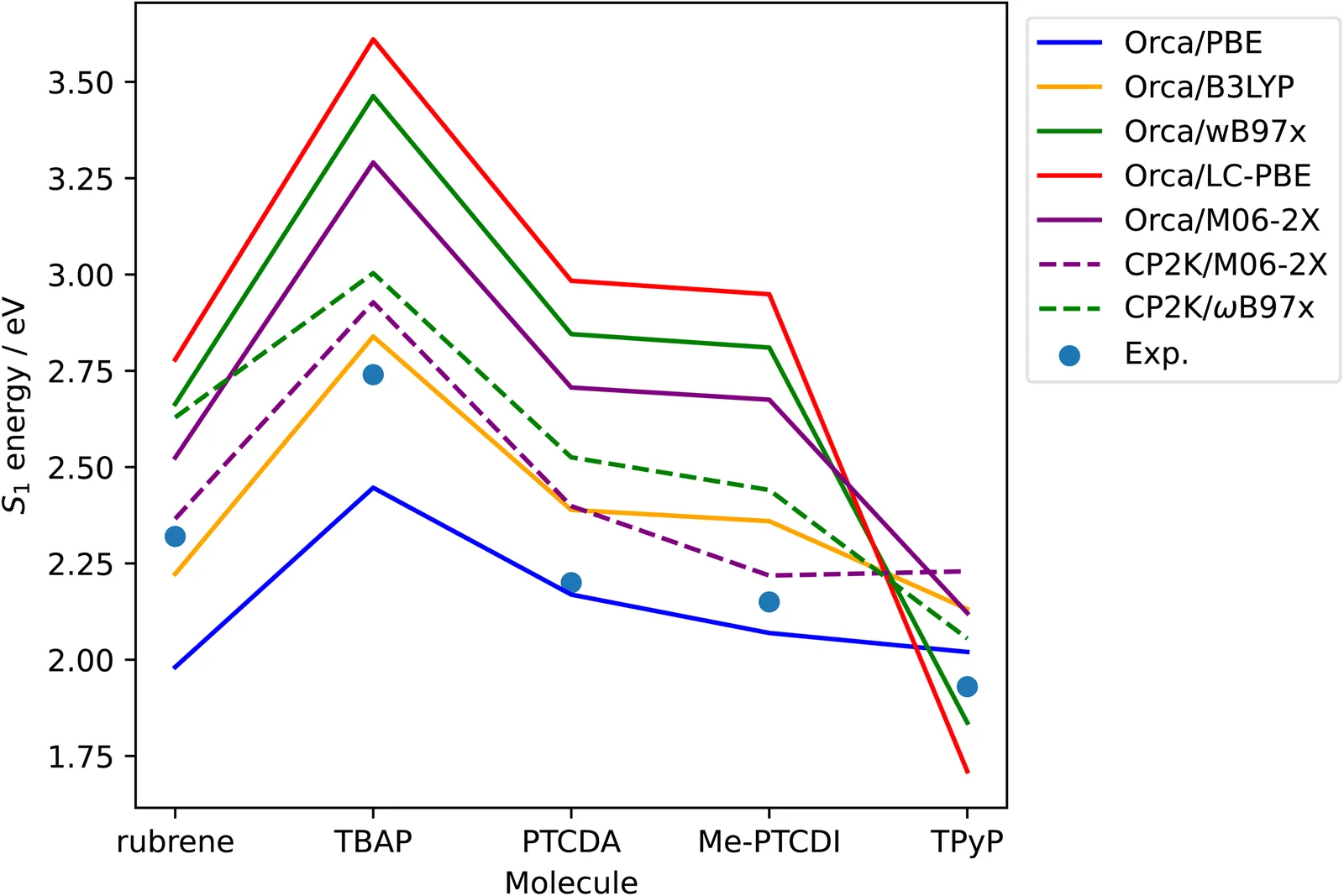
Comparison of the S_1_ energies for five organic molecular crystals calculated by periodic TD-DFPT (M06-2X and *ω*B97x in CP2K^75^) with molecular TD-DFT (using four functionals: B3LYP, *ω*B97x, LC-PBE and M06-2X in Orca^38,81^). The experimental optical gap deduced from thin-film UV-visible absorption spectra is also plotted for reference.^89–92^

We now compare the S_1_ energies predicted by periodic TD-DFPT to those obtained using molecular TD-DFT calculations, as shown in Fig. 3. Five different exchange–correlation functionals: PBE,^70^ B3LYP,^86^*ω*B97x,^78^ LC-PBE^87^ and M06-2X^77^ were tested to ascertain the functional dependence of the excitation energies. These results are presented in Fig. 3. The molecular calculations using range-separated and hybrid meta-GGA *ω*B97x (root mean squared error, RMSE = 0.55 eV), LC-PBE (RMSE = 0.67 eV) and M06-2X (RMSE = 0.43 eV) functionals all perform worse than periodic M06-2X (RMSE = 0.18 eV) and periodic *ω*B97x (RMSE = 0.27 eV) at predicting S_1_ and generally overestimate S_1_ energies by a large margin (apart from for TPyP). On the other hand, when using the hybrid GGA B3LYP functional (RMSE = 0.17 eV), molecular calculations perform on a par with the periodic calculations and closely reproduce the experimental S_1_ energies. The reason that the range-separated and hybrid meta-GGA functionals, usually considered to be higher in accuracy, perform worse than B3LYP is due to the well known cancellation of two types of errors in the molecular calculations: B3LYP tends to systematically underestimate the HOMO–LUMO/band gap and thus the excitation energies, but conversely, excitation energies tend to be systematically lower in the solid state compared to the gas phase due to a combination of exciton delocalisation and electrostatic polarisation effects of the solid medium.^93–95^

To assess the relative computational cost of periodic *versus* molecular calculation of the optical gap, a periodic calculation (TD-DFPT using M06-2X/DZVP-MOLOPT in CP2K on a 608 atom supercell of PTCDA) was compared with a molecular calculation (TD-DFT B3LYP/def2-TZVP in Orca on a single PTCDA molecule) both calculating 10 singlet roots. The former was run using 512 processes on Archer2^69^ and took 2 hours 47 minutes and the latter was run on a modern sixteen-core desktop computer and took 5 minutes. Taking into account the total CPU time (∼1420 hours and 1.33 hours respectively), the molecular calculation is about three orders of magnitude less computationally expensive.

One caveat is that molecular calculations should only be relied on for systems where S_1_ is a local (Frenkel) excitation. In the case where S_1_ is in fact an intermolecular charge transfer state (Wannier–Mott exciton), the single-molecule model for the optical gap will certainly fail. This rules out investigation of systems such as co-crystals where low energy CT states are common.^96^ Apart from some notable exceptions that are hotly debated in the literature,^97,98^ the majority of single-component molecular crystals exhibit Frenkel-like S_1_ states,^99^ so the molecular picture should still be sufficiently generalisable. Davydov splitting can shift the S_1_ energy from that of a single Frenkel excitation and is also not captured by single-molecule calculations,^43,99^ however even in the case of strong Davydov splitting, molecular calculations should give a rough estimate figure for the S_1_ energy. Instead of employing gas-phase molecular calculations, a common approach is to mimic the polarisation effects of the crystal environment by employing polarisable continuum (PCM) solvation models in molecular DFT, correcting the excited state energies.^93,99,100^ We refrain from using this approach here for the sake of simplicity as we already see reasonable accuracy for gas-phase B3LYP.

### Reduction and oxidation potentials

The vertical IPs and EAs were calculated for the organic crystals using HSE06 band structures and the vacuum slab surface dipole method as described in the previous section. To complement these results, molecular gas-phase DFT calculations with a range of functionals (PBE, B3LYP, *ω*B97x, LC-PBE, M06-2X) were employed to estimate the IP (by taking the negative of the HOMO energies) and the EA (by taking the negative of the LUMO energies) again in the vertical approximation.^70,77,78,86,87^ These results are shown in Tables 2 and 3 and Fig. 4 and 5. We also draw reference to the oxidation potential of water (5.65 V) and reduction potential of protons (4.42 eV) to compare the predicted driving force for HER and OER for the molecular crystals.

**Table 2:** DFT-derived vertical ionisation potentials for the five organic molecular crystals calculated from the vacuum-corrected HSE06 VBM of the crystal structures (HSE PAW). Also shown are IPs calculated directly from HOMO energies of gas-phase single-molecule PBE, B3LYP and *ω*B97x calculations. Literature experimental values from thin-film photoelectron spectra are also shown where available for comparison with their corresponding literature reference

Molecule	IP (exp.)/eV	Ref.	IP (HSE06 PAW)/eV	IP (PBE)/eV	IP (B3LYP)/eV	IP (*ω*B97x)/eV
Rubrene	4.85	101	4.34	4.46	4.81	6.83
TBAP	—	—	5.49	5.19	5.47	7.44
PTCDA	6.62	100	6.66	6.24	6.63	8.65
Me-PTCDI	6.60	100	6.06	5.72	6.12	8.14
TPyP	—	—	6.08	5.26	5.57	7.34

**Table 3:** DFT-derived vertical electron affinities for the five organic molecular crystals calculated from the vacuum-corrected HSE06 CBM of the crystal structures (HSE PAW). Also shown are EAs calculated directly from LUMO energies of gas-phase single-molecule PBE, B3LYP and *ω*B97x calculations. Literature experimental values from thin-film photoelectron spectra are also shown where available for comparison with their corresponding literature reference

Molecule	EA (exp.)/eV	Ref.	EA (HSE06 PAW)/eV	EA (PBE)/eV	EA (B3LYP)/eV	EA (*ω*B97x)/eV
Rubrene	—	—	2.72	2.98	2.27	0.63
TBAP	—	—	3.24	3.34	2.61	1.00
PTCDA	4.10	100	4.83	4.79	4.19	2.66
Me-PTCDI	3.95	100	4.50	4.28	3.69	2.18
TPyP	—	—	3.97	3.52	2.91	1.62

**Fig. 4:**
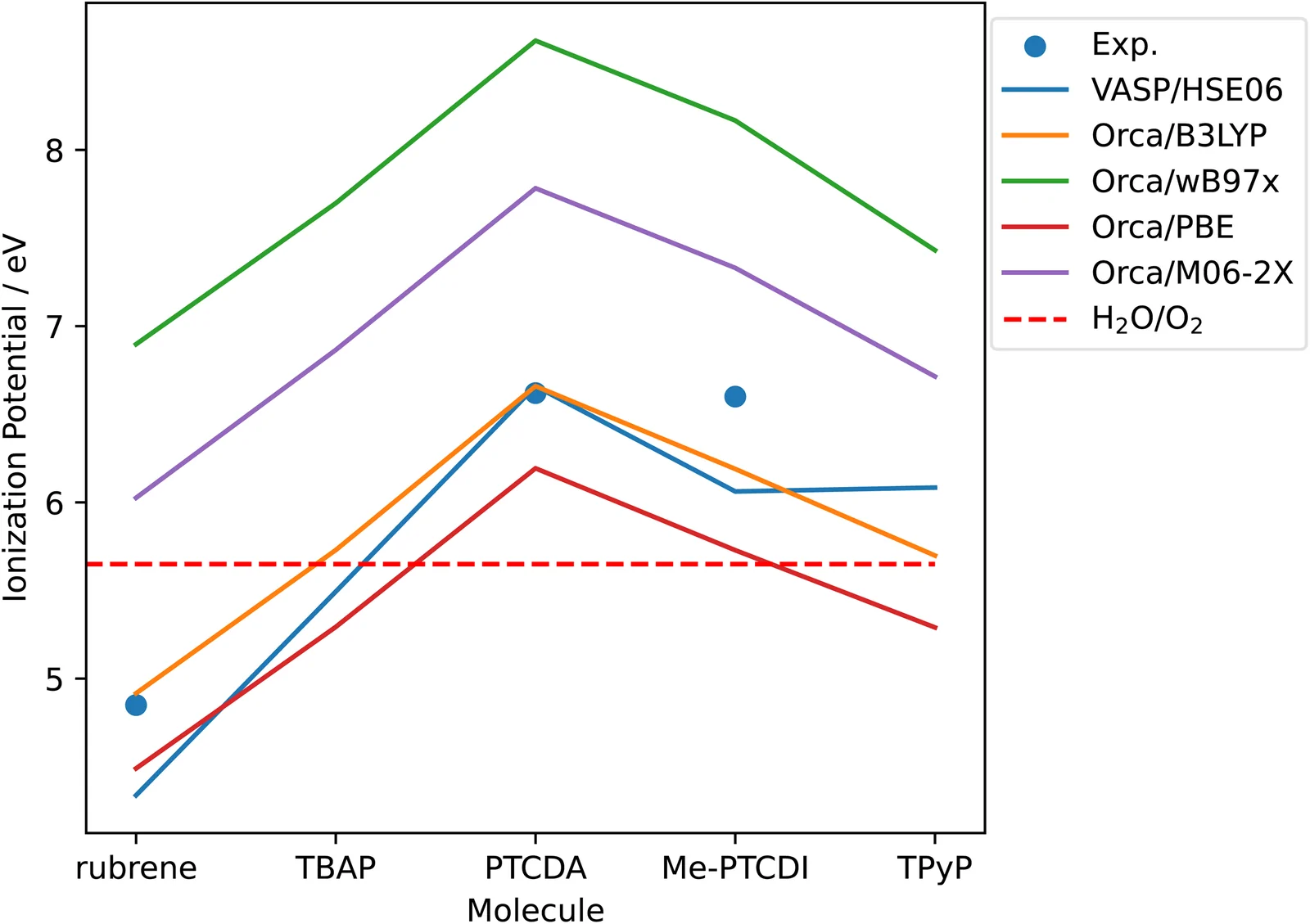
DFT-derived vertical IPs for the five organic molecular crystals calculated using the vacuum-corrected HSE06 VBM of the crystal structures and calculated directly from HOMO energies of gas-phase single-molecule B3LYP, *ω*B97x, LC-PBE and M06-2X calculations. Literature data from thin-film photoelectron spectra are also shown.^100,101^

**Fig. 5:**
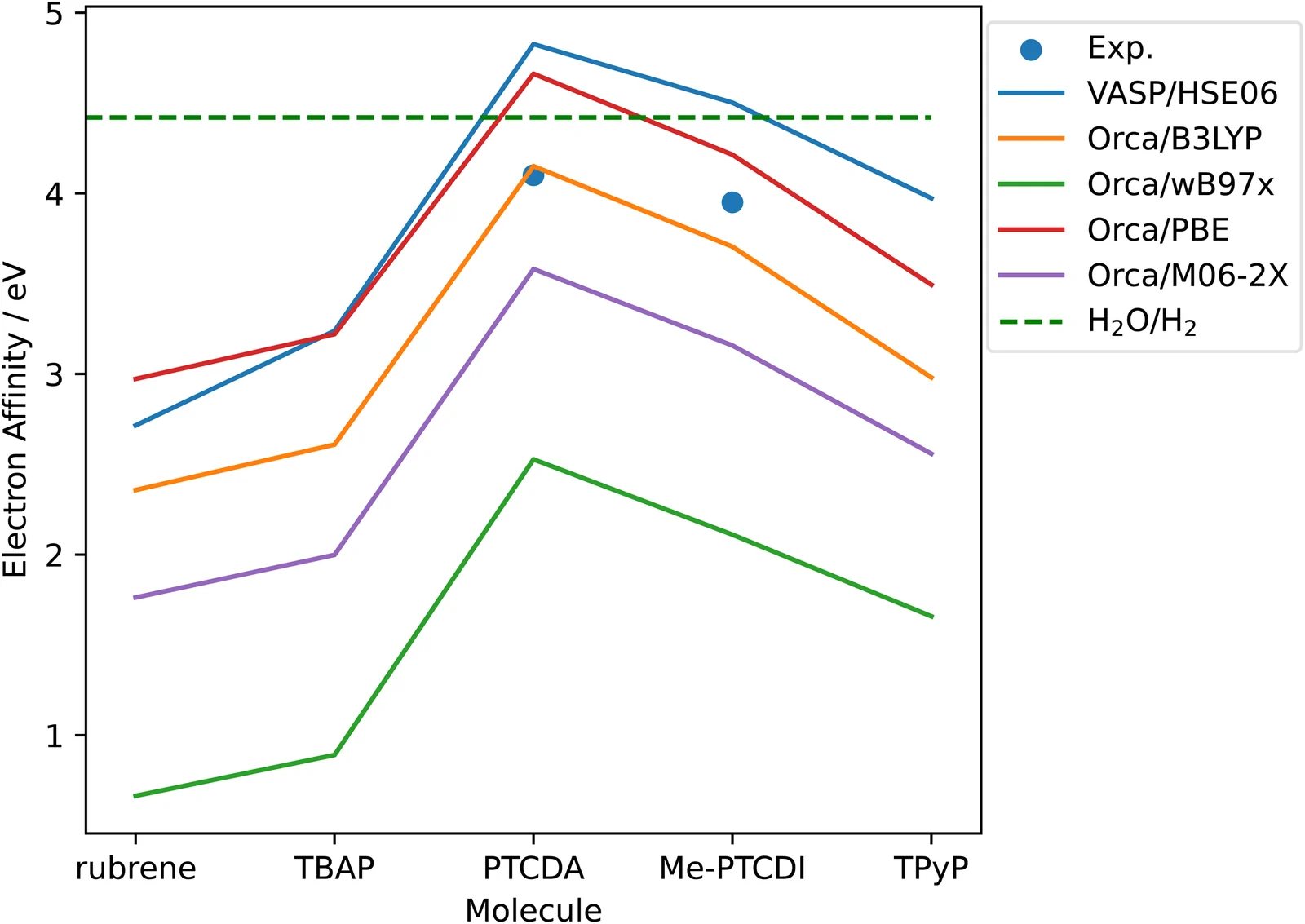
DFT-derived vertical EAs for the five organic molecular crystals calculated using the vacuum-corrected HSE06 CBM of the crystal structures and calculated directly from LUMO energies of gas-phase single-molecule B3LYP, *ω*B97x, LC-PBE and M06-2X calculations. Literature data from thin-film photoelectron spectra are also shown.^100^

The agreement between the HSE06 calculated IP with experiment is very good for PTCDA, with an error of less than 0.1 eV, however for rubrene and Me-PTCDI the agreement is less good, with errors of 0.51 eV and 0.54 eV respectively (Table 2). The RMSE of 0.43 eV for the IP compared to experiment is consistent with experimental errors of previously reported calculated IPs for similar molecular crystals (∼0.4 eV)^93,100^ however, our sample size is very small (5 molecules in this work compared to 11 and 13 in ref. 93 and 100 respectively). The HSE06 calculated and experimental IPs follow the same trend as experiment, albeit there are only 3 experimental data points, so comparison is somewhat limited. The agreement between HSE06 calculated EAs and experiment is slightly worse than for the IP, with errors of 0.73 and 0.55 eV for PTCDA and Me-PTCDI respectively (Table 3). Again, comparison with experiment is limited due to the lack of thin-film data. Based on HSE06 calculations, PTCDA has the highest IP and rubrene has the lowest IP. PTCDA also has the highest EA and rubrene the lowest EA.

The HSE06 calculated values in Tables 2 and 3 and Fig. 4 and 5 predict that while rubrene should be capable of HER, it does not have a sufficient oxidation potential (4.34 eV calculated, 4.85 eV experiment) to drive OER. The HSE06 IP of TBAP lies 0.16 eV below the water oxidation potential, however this difference is within the RMSE of 0.43 eV so OER is conceivable and its EA of 3.24 eV is sufficiently below the proton reduction potential so HER should be possible. OER should be feasible in PTCDA due to its high IP (both calculated and experimental) however its EA of 4.83 eV is 0.41 eV above the proton reduction potential so proton reduction seems unlikely. Although the experimental EA of PTCDA of 4.10 eV is below the proton reduction potential and would suggest HER to be viable,^102^ experimental evidence suggests otherwise as the rate of sacrificial hydrogen evolution is effectively zero in pure PTCDA.^28,29^ OER should also be feasible in Me-PTCDI with its high IP, and while the HSE06 predicted EA of Me-PTCDI lies 0.08 eV above the hydrogen reduction potential, this is within the margin of error, so HER is conceivable. HSE06 predicts both OER and HER to be feasible in TPyP with an IP of 6.08 eV – above the water oxidation potential, and an EA of 3.97 eV – below the proton reduction potential.

Considering gas-phase molecular calculations for the IP and EA, we again notice that energies calculated with B3LYP are the most accurate overall for all the molecular calculations. This is again likely due to a cancellation of errors in molecular orbital energies, where stabilising polarisation effects in the solid medium are neglected yet there is a systematic underestimation of the HOMO/LUMO gap when using the B3LYP which compensates for this.^93–95^ Again we elect not to use the PCM and we also approximate the IPs and EAs using Koopman's theorem rather than taking energies of separate neutral and charged species for the sake of simplicity. When compared to similar studies of PTCDA and Me-PTCDI using the two-point approach and applying PCM,^93,100^ employing gas-phase B3LYP HOMO and LUMO energies gives similar accuracy for the IPs and EAs.

Gas-phase B3LYP proves to be accurate with respect to higher level periodic HSE06 and experimental data for calculating the IP/EA of the molecular crystals studied here. Comparing the computational cost of periodic HSE06 against gas-phase B3YLP, for PTCDA the total compute time taken for the former including the band structure and slab calculations was 4370 core hours (on Archer2^69^) and for the latter was 0.45 core hours (on a modern desktop computer) making the molecular calculation four orders of magnitude faster. Additionally, cutting the vacuum slabs had to be done manually and added significant time to the process. In summary, for these molecular crystals, computationally inexpensive molecular DFT calculations are sufficient to predict whether HER and OER are thermodynamically feasible in these materials, and therefore are suitable for large-scale computational screening.

## Conclusions

We have addressed computational descriptors covering the fundamental electronic processes in photocatalytic overall water splitting for a series of organic molecular crystals by both periodic and molecular density functional theory calculations. The optical gaps, IP and EA were calculated for molecular crystals of one acene (rubrene), two perylene-based molecules (PTCDA, Me-PTCDI), one pyrene-based molecule (TBAP) and one porphyrin (TPyP), where we find reasonably good agreement between periodic DFT and experiment for all properties. DFT calculated IP/EA and experimental values taken from the literature both predict that rubrene does not have a sufficiently high oxidation potential to drive OER, and PTCDA has an reduction potential too high to drive HER. DFT calculations suggest that TBAP, Me-PTCDI and TPyP have the correct hole and electron potentials to drive both OER and HER, suggesting that from an energetic perspective, they would be the most promising molecular starting materials of those tested for the design of a single-component OWS photocatalyst. Moreover, we find that gas-phase molecular calculations with hybrid-GGA functionals such as B3LYP offer comparable accuracy to much more computationally demanding periodic DFT calculations when predicting the optical gap and IP/EA of molecular crystals. We have shown, by comparison to experimental data and the results of high level solid state DFT calculations, that gas-phase molecular DFT/TD-DFT calculations using the B3LYP functional offer sufficient accuracy for screening the optoelectronic properties of molecular crystals for photocatalytic water splitting applications and with at least three orders of magnitude reduced computational cost compared to periodic DFT. In future work, the hopping carrier transport model for organic molecular crystals could be considered, investigating to what extent the computational cost of the hopping-like model could be reduced whilst still predicting qualitative trends in charge transport rates that are relevant for OWS.

## Author contributions

The paper was conceived by JDG with help from KEJ, HW and AIC. Computational work and analysis of results was carried out by JDG. The majority of the paper was initially written by JDG. KEJ and DGM assisted with revising the paper. HW and AIC advised on photocatalytic OWS and organic materials to investigate. DGM and JN advised on the theoretical discussion of charge transport.

## Conflicts of interest

There are no conflicts of interest to declare.

## Supplementary Material

FD-268-D6FD00050A-s001

## Data Availability

The research data for this article can be found herein and in the supplementary information (SI). Supplementary information: details of the crystal structures used in this work including the lattice constants of relaxed primitive cells, supercells and slabs used in addition to the HSE06 band structures of all molecular crystals studied. See DOI: https://doi.org/10.1039/d6fd00050a.
