# Supplementary material for: A computational study of organic molecular crystals for photocatalytic water splitting
Source: Faraday Discuss. 2026 Jul 20;268:110–26. doi: 10.1039/d6fd00050a (PMC13719147; doi:10.1039/d6fd00050a)
Supplement: Supplementary file 1 [file FD-268-D6FD00050A-s001.pdf]

Supplementary Information (SI) for Faraday Discussions.
This journal is © The Royal Society of Chemistry 2026

Supplementary information for "A Computational Study of Organic Molecular Crystals for Photocatalytic Water Splitting"

19Th March 2026

James D. Green,[*a] Hong Wang[b], Andrew I. Cooper[b], Daniel G. Medranda[c], Jenny Nelson[c] and Kim E. Jelfs[a]

a. Department of Chemistry, Molecular Sciences Research Hub, Imperial College London, London W12 0BZ, UK
b. Department of Chemistry, University of Liverpool, Liverpool L69 7ZD, United Kingdom
c. Department of Physics, Imperial College London, London, UK

* E-mail: j.green@ic.ac.uk

S1. Lattice Constants

Table S1 – Lattice constants for X-ray crystal structures of the five molecular crystals studied taken from the Cambridge Structural Database[1].

Molecule	Ref code	a / Å	b / Å	c / Å	α / deg.	β / deg.	γ / deg.
rubrene	QQQCIG01	7.1841	14.4333	26.8977	90	90	90
TBAP	MUDSIQ	29.094	28.614	3.9286	90	91.872	90
PTCDA	SUWMIG02	3.7036	12.0135	17.1613	90	93.266	90
Me-PTCDI	DICPAG	3.8741	15.5802	14.5972	90	97.652	90
TPyP	COWSIR	21.0421	6.88801	21.75601	90	90.3701	90

Table S2 - Lattice constants for relaxed cells (PBE-D3 in VASP[2]) in conventional setting.

Molecule	a / Å	b / Å	c / Å	α / deg.	β / deg.	γ / deg.
rubrene	14.106	7.166	26.900	90.000	90.000	90.000
TBAP	28.973	28.509	3.804	90.000	92.441	90.000
PTCDA	3.674	11.978	16.959	90.000	93.008	90.000
Me-PTCDI	3.823	15.511	14.382	90.000	98.901	90.000
TPyP	20.592	6.875	21.348	90.000	91.550	90.000

Table S3 - Lattice constants for relaxed cells (PBE-D3 in VASP[2]) in primitive setting (if different to conventional cell).

Molecule	a / Å	b / Å	c / Å	α / deg.	β / deg.	γ / deg.
rubrene	13.919	13.919	14.106	90.000	90.000	29.834
TBAP	20.324	20.324	3.804	91.740	91.740	89.076
TPyP	10.855	10.855	21.348	88.530	88.530	36.923

Table S4 - Lattice constants for supercells used for TD-DFT calculations of optical gaps of the five molecular crystals studied (in CP2K[3,4], gamma point only).

Molecule	a / Å	b / Å	c / Å	α / deg.	β / deg.	γ / deg.
rubrene	14.332	26.900	14.106	90.000	90.000	90.000
TBAP	20.324	20.324	15.216	91.740	91.740	89.076
PTCDA	14.697	23.956	16.959	90.000	93.008	90.000
Me-PTCDI	15.292	15.511	14.382	90.000	98.901	90.000
TPyP	21.709	21.709	21.348	88.530	88.530	36.923

*Table S5 - Lattice constants for vacuum slabs cut from the relaxed conventional cells used for IP/EA calculations of the five molecular crystals studied.**

Molecule	a / Å	b / Å	c / Å	α / deg.	β / deg.	γ / deg.
rubrene	7.166	26.900	70.528	90.000	90.000	90.000
TBAP	28.973	28.509	68.473	90.000	92.441	90.000
PTCDA	16.959	11.978	66.137	90.000	86.992	90.000
Me-PTCDI	14.382	15.511	61.169	90.000	81.099	90.000
TPyP	20.592	6.875	64.043	90.000	91.550	90.000

**For PTCDA and Me-PTCDI, β is different from the conventional cell as the a and c crystallographic axes were interchanged so β →− β. The transformed conventional cell and the original conventional cell are geometrically equivalent in this process and $\beta_{transformed\ cell} + \beta_{original\ cell} = 180$ degrees.*

S2. DFT (PBE-D3) relaxed crystal structures

Table S6 - Parameters used for cell relaxations in VASP[2] and details of the structural comparisons in Mercury 2024.1.0 [5] of the relaxed structures with x-ray crystal structures taken from the CCDC for the five molecular crystals studied.

Molecule	Atoms in prim. cell	Method	ENCUT / eV	k-points / a b c	Compared to	Matching molecules	RMSD / Å
Rubrene	140	PBE-D3	650	2 2 2	QQQCIG01	30 of 30	0.14
TBAP	78	PBE-D3	650	3 3 3	MUDSIQ	30 of 30	0.23
PTCDA	76	PBE-D3	650	3 3 3	SUWMIG02	30 of 30	0.08
Me-PTCDI	92	PBE-D3	650	3 3 3	DICPAG	30 of 30	0.13
TPyP	148	PBE-D3	650	2 2 2	COWSIR	30 of 30	0.27

S3. Parameters used in periodic TD-DFT calculations

Table S7 - Settings used in periodic TD-DFT calculations in CP2K[3,4] for all five molecular crystals studied. Input files were generated using Multiwfn[6,7] which suggested these values by default.

**Functional**	M06-2X	ωB97x
**Valence basis**	DZVP-MOLOPT	DZVP-MOLOPT
**Pseudopotential**	GTH-PBE	GTH-PBE
**Density fitting**	ADMM	ADMM
**Grid cutoff / eV**	350	350
**Relative cutoff / eV**	50	50
**Interaction potential**	TRUNCATED	MIX_CL_TRUNC
**Interaction pot. cutoff radius / Å**	6	6
**HF composition**	0.54	1
**Scaling (Coulomb part)**	n/a	0.157706
**Scaling (Long-range part)**	n/a	0.842294
**ω**	n/a	0.3

S4. K-point meshes used for vacuum slabs

TBAP and Me-PTCDI used gamma point only. Rubrene, PTCDA and TPyP used gamma-centred meshes of 3x1x1, 1x2x1 and 1x3x1 k-points in a, b and c cell directions respectively.

S5. Computational band structures

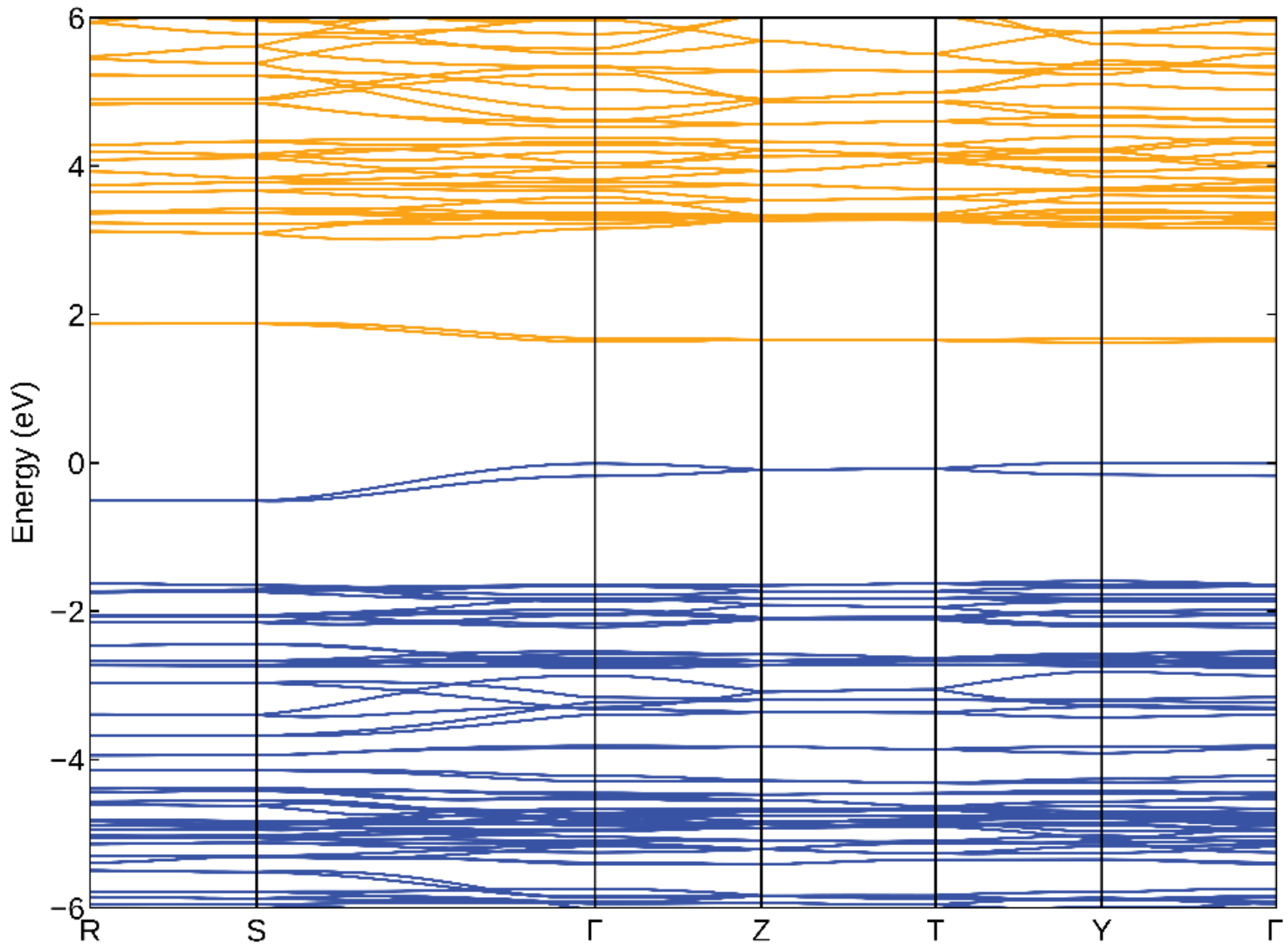

Figure S1 - DFT (HSE06 in VASP[2]) band structure of rubrene (primitive cell) using gamma-centred mesh of 3x3x3 k-points along a, b and c lattice directions and ENCUT of 650 eV.

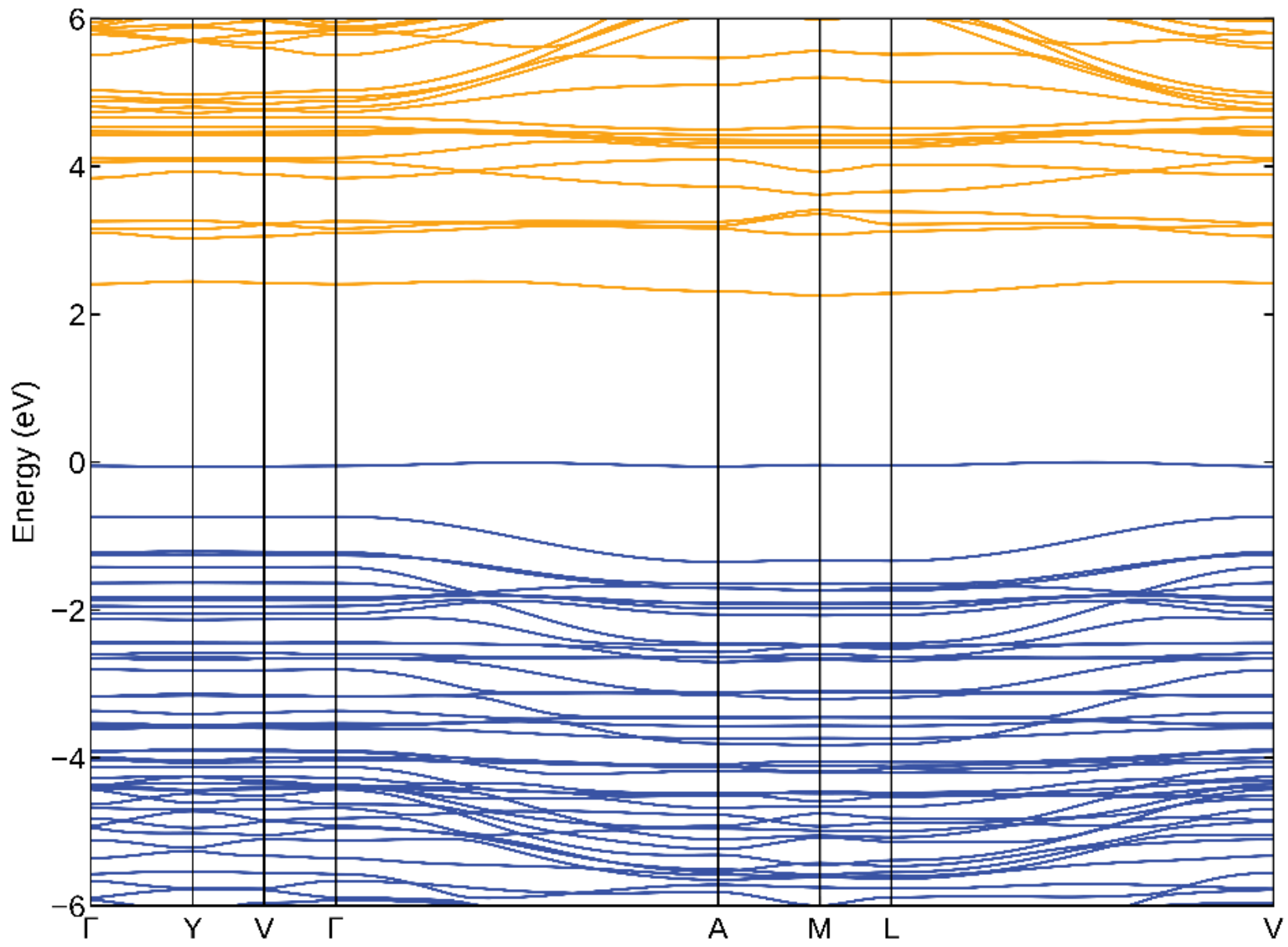

Figure S2 - DFT (HSE06 in VASP[2]) band structure of TBAP (primitive cell) using gamma-centred mesh of 2x2x4 k-points along a, b and c lattice vectors and ENCUT of 650 eV.

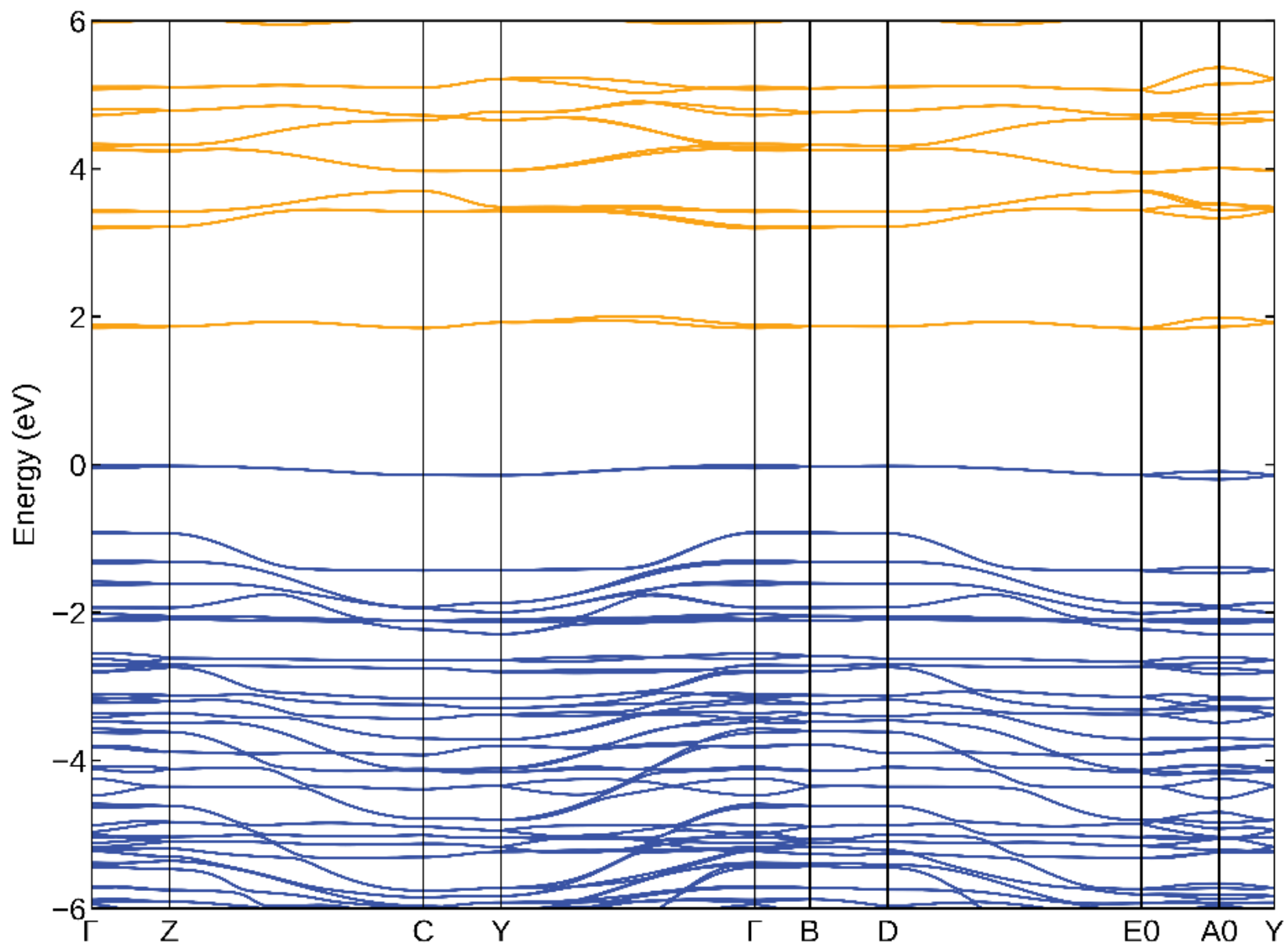

Figure S3 - DFT (HSE06 in VASP[2]) band structure of PTCDA using gamma-centred mesh of 4x2x1 k-points along a, b and c lattice vectors and ENCUT of 650 eV.

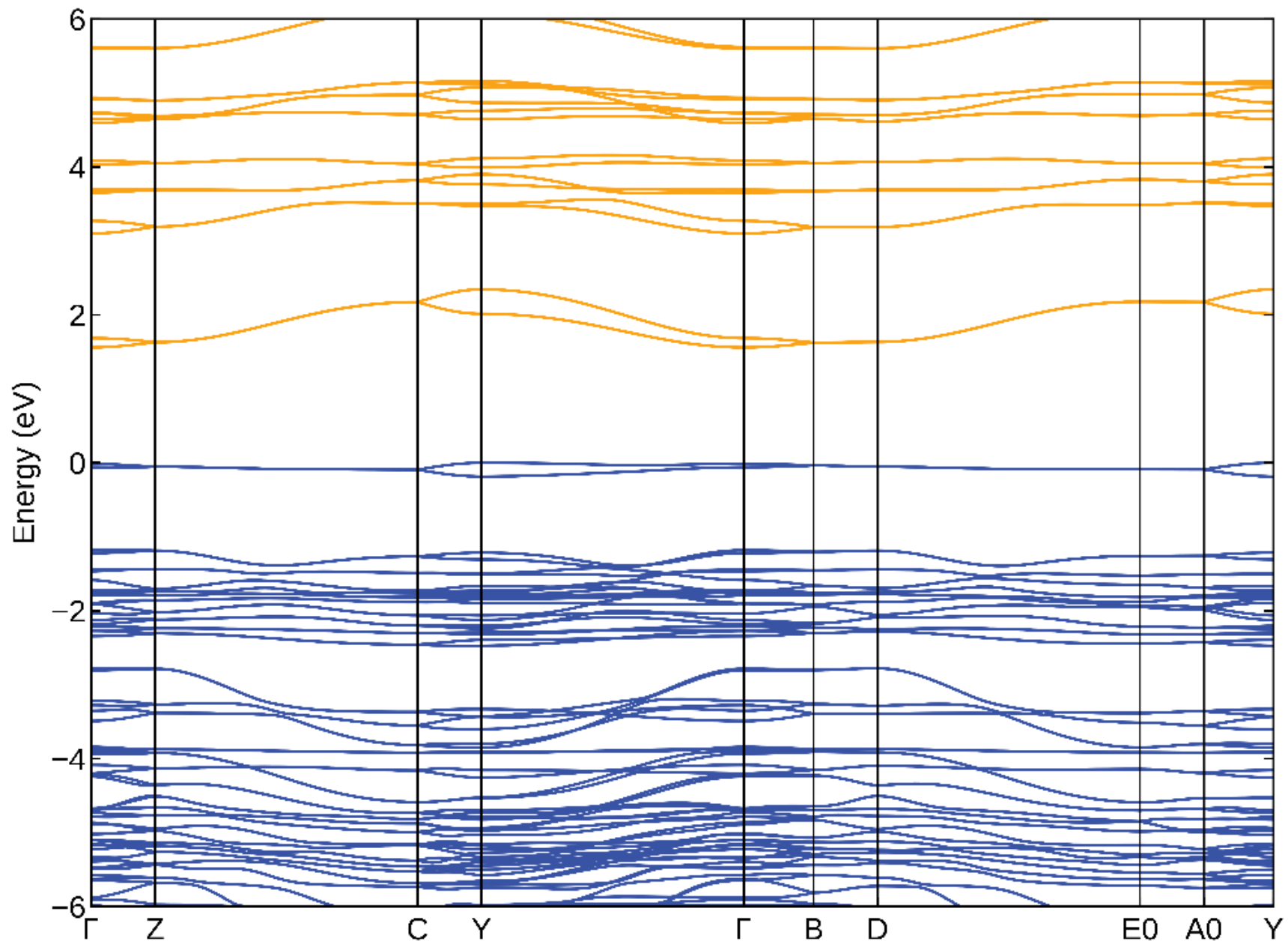

Figure S4 - DFT (HSE06 in VASP[2]) band structure of Me-PTCDI using gamma-centred mesh of 4x2x2 k-points along a, b and c lattice vectors and ENCUT of 650 eV.

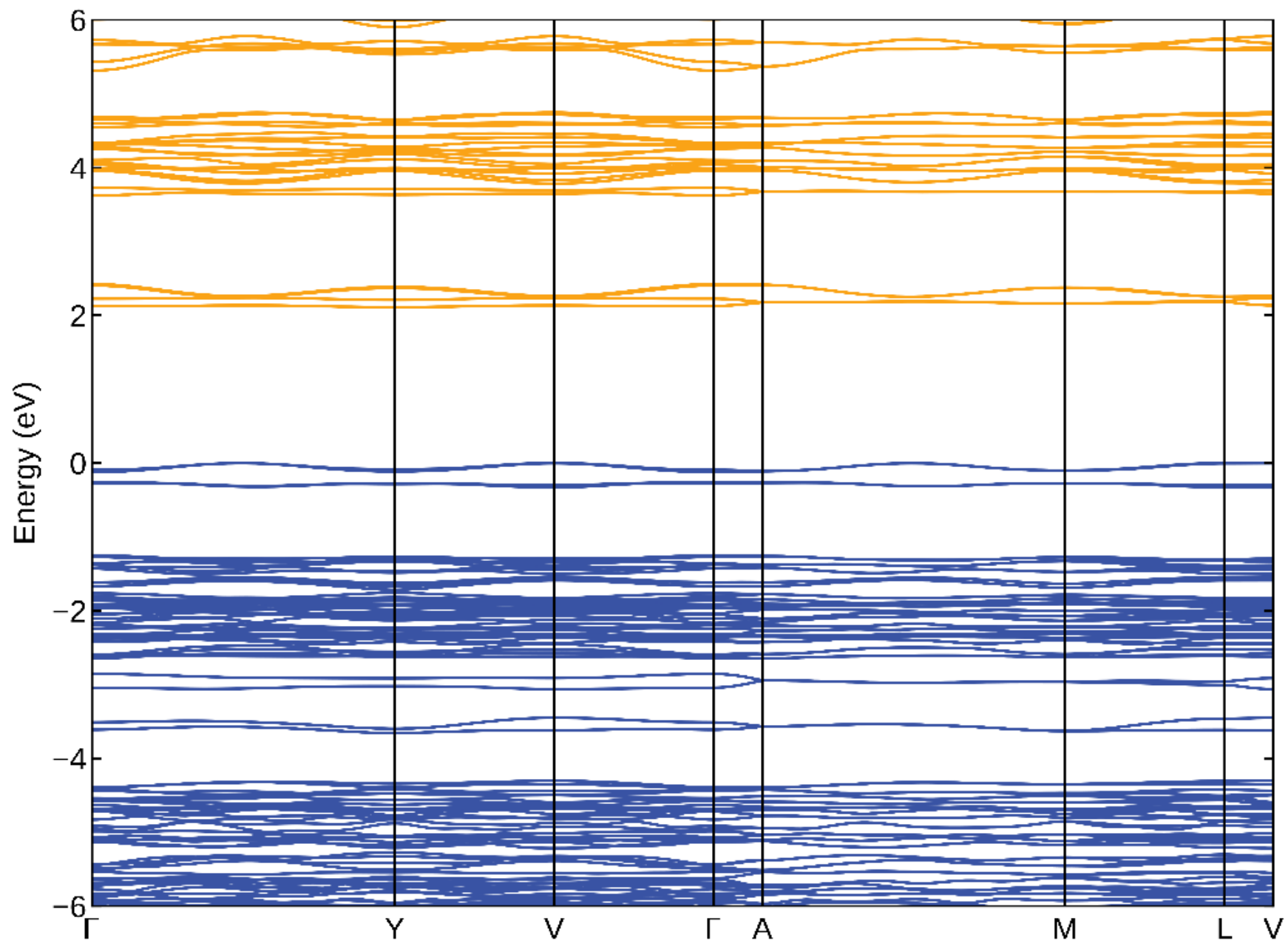

Figure S5 - DFT (HSE06 in VASP[2]) band structure of TPyP (primitive cell) using gamma-centred mesh of 2x2x1 k-points along a, b and c lattice vectors and ENCUT of 650 eV.

References

1 C. R. Groom, I. J. Bruno, M. P. Lightfoot and S. C. Ward, *Acta Crystallographica Section B*, 2016, **72**, 171–179.

2 G. Kresse and J. Furthmüller, *Phys. Rev. B*, 1996, **54**, 11169–11186.

3 J. Hutter, M. Iannuzzi, F. Schiffmann and J. VandeVondele, *WIREs Computational Molecular Science*, 2014, **4**, 15–25.

4 T. D. Kühne, M. Iannuzzi, M. Del Ben, V. V. Rybkin, P. Seewald, F. Stein, T. Laino, R. Z. Khaliullin, O. Schütt, F. Schiffmann, D. Golze, J. Wilhelm, S. Chulkov, M. H. Bani-

Hashemian, V. Weber, U. Borštnik, M. Taillefumier, A. S. Jakobovits, A. Lazzaro, H. Pabst, T. Müller, R. Schade, M. Guidon, S. Andermatt, N. Holmberg, G. K. Schenter, A. Hehn, A. Bussy, F. Belleflamme, G. Tabacchi, A. Glöß, M. Lass, I. Bethune, C. J. Mundy, C. Plessl, M. Watkins, J. VandeVondele, M. Krack and J. Hutter, *The Journal of Chemical Physics*, 2020, **152**, 194103.
5 C. F. Macrae, I. Sovago, S. J. Cottrell, P. T. A. Galek, P. McCabe, E. Pidcock, M. Platings, G. P. Shields, J. S. Stevens, M. Towler and P. A. Wood, *Journal of Applied Crystallography*, 2020, **53**, 226–235.
6 T. Lu, *The Journal of Chemical Physics*, 2024, **161**, 082503.
7 T. Lu and F. Chen, *Journal of Computational Chemistry*, 2012, **33**, 580–592.
